# Ran GTPase: A Key Player in Tumor Progression and Metastasis

**DOI:** 10.3389/fcell.2020.00345

**Published:** 2020-05-26

**Authors:** Zied Boudhraa, Euridice Carmona, Diane Provencher, Anne-Marie Mes-Masson

**Affiliations:** ^1^Centre de Recherche du Centre Hospitalier de l’Université de Montréal (CRCHUM), Montreal, QC, Canada; ^2^Institut du Cancer de Montréal (ICM), Montreal, QC, Canada; ^3^Division of Gynecologic Oncology, Université de Montréal, Montreal, QC, Canada; ^4^Department of Medicine, Université de Montréal, Montreal, QC, Canada

**Keywords:** Ran GTPase, cancer, metastasis, survival, proliferation

## Abstract

Ran (Ras-related nuclear protein) GTPase is a member of the Ras superfamily. Like all the GTPases, Ran cycles between an active (GTP-bound) and inactive (GDP-bound) state. However, Ran lacks the CAAX motif at its C-terminus, a feature of other small GTPases that ensures a plasma membrane localization, and largely traffics between the nucleus and the cytoplasm. Ran regulates nucleo-cytoplasmic transport of molecules through the nuclear pore complex and controls cell cycle progression through the regulation of microtubule polymerization and mitotic spindle formation. The disruption of Ran expression has been linked to cancer at different levels – from cancer initiation to metastasis. In the present review, we discuss the contribution of Ran in the acquisition of three hallmarks of cancer, namely, proliferative signaling, resistance to apoptosis, and invasion/metastasis, and highlight its prognostic value in cancer patients. In addition, we discuss the use of this GTPase as a therapeutic target in cancer.

## Introduction

Ran (Ras-related nuclear protein) is a member of the RAS superfamily of small GTPases. This superfamily is subdivided into five families: Ras (36 members), Rho (20 members), ARF (27 members), Rab (61 members), and Ran (one member) (Wennerberg et al., 2005). Ran is unique among other GTPases owing to its acidic tail at the C-terminus. Furthermore, unlike the other GTPases, Ran lacks the CAAX motif, a membrane-anchoring peptide (Scheffzek et al., 1995; Seewald et al., 2003; Monecke et al., 2009). In fact, while other GTPases are often cytoplasmic or associated with subcellular membranes, Ran GTPase is shared between the nucleus and the cytoplasm (Matchett et al., 2014). Structurally, Ran is a protein composed of 216 amino acids with a molecular weight of approximately 25 kDa. It contains a central G domain (the GTP-binding and hydrolysis domain) comprised of residues 8 to 210, forming a six-stranded β-sheet surrounded by five α-helices (Scheffzek et al., 1995). This G domain contains a phosphate-binding loop or the P-loop (17-GDGGTGKT-24) that, together with a Mg^2+^ ion, interacts with the oxygens of α, β, and γ phosphates (from GTP or GDP) to stabilize nucleotide binding (Scheffzek et al., 1995). Furthermore, the G domain displays two critical motifs, switch I (residues 32–45) and switch II (residues 66–79), that upon nucleotide exchange undergo a conformational change, allowing Ran to interact or dissociate with its partners (Scheffzek et al., 1995; Stewart et al., 1998; Chook and Blobel, 1999; Vetter et al., 1999a, b). Besides its G domain, Ran has a unique acidic C-terminus tail (211-DEDDDL-216) (Scheffzek et al., 1995). In Ran’s inactive state, this tail is unstructured and in contact with a basic patch on the surface of Ran (residues 139–142) to stabilize its GDP conformation (Richards et al., 1995; Scheffzek et al., 1995). Following activation (exchange from GDP to GTP-bound state), switches I and II undergo a dramatic conformational change, leading to the shift of this C-terminus tail out from the G domain and making the GTPase available for interaction with several partners (Chook and Blobel, 1999; Knyphausen et al., 2015). Several studies have investigated Ran motifs engaged in the interaction of Ran with its partners. It appears that while switch I and the basic patch of Ran are involved in the interaction with importins and exportins (Steggerda and Paschal, 2002; Guttler and Gorlich, 2011), the C-terminus tail is involved in the interaction with other proteins such as RanBP1, RanBP2, and the newly identified partner, RhoA (Macara, 1999; Villa Braslavsky et al., 2000; Seewald et al., 2002; Geyer et al., 2005; Zaoui et al., 2019). Like all the GTPases, Ran activity relies on a specific guanine nucleotide exchange factor [Regulator of Chromosome Condensation 1 (RCC1), also known as RanGEF] that promotes the GTP loading of Ran by interacting with the P-loop and switch II (Renault et al., 2001) and a GTPase-activating protein [Ran GTPase Activating Protein 1 (RanGAP1)] that participates in the hydrolysis of GTP to GDP by interacting with the P-loop, switch I, and switch II (Seewald et al., 2002; Joseph, 2006). Since these GTP loading and hydrolyzing partners are, respectively, localized in the nucleus and the cytoplasm, this creates a Ran-GTP gradient across the nuclear envelope (NE) with a higher concentration of Ran-GTP in the nucleus than in the cytoplasm (Matchett et al., 2014).

Ran performs two major and distinct cellular functions. During interphase, Ran regulates nucleo-cytoplasmic transport of molecules through the nuclear pore complex (Sorokin et al., 2007; Stewart, 2007). At mitosis, Ran controls cell cycle progression through the regulation of the mitotic spindle and NE formation (Matchett et al., 2014). The Ran-GTP/GDP cycle is regulated by several proteins (Ohtsubo et al., 1989; Bischoff et al., 1995; Matunis et al., 1996; Mahajan et al., 1997) that are involved in both major physiological functions of Ran through different gradients (Kalab et al., 2002). The traffic of bioactive molecules between the nucleus and the cytoplasm occurs through nuclear pore complexes (NPCs), which are formed by a set of proteins called nucleoporins, embedded in the NE (Watson, 1954). These NPCs form aqueous channels with a diameter of 40–50 nm connecting the cytoplasmic and nuclear compartments. However, while small molecules may traffic passively, these channels hinder the diffusion of larger molecules (diameter greater than 5 nm which corresponds to proteins larger than approximately 30 kDa) (Mohr et al., 2009). The traffic of these proteins requires an active transport mechanism which involves shuttling adapter molecules and nuclear transport receptors (NTRs) as well as Ran-GTP that feeds the metabolic energy required for this process (Steggerda and Paschal, 2002). Ran-GTP-dependent receptors are the largest NTR class comprised of 21 members in mammals. These receptors share an N-terminal Ran-binding domain and are categorized into importins and exportins. They recruit cargo proteins with a nuclear localization signal (NLS) or a nuclear export signal (NES), respectively (Rexach and Blobel, 1995; Gorlich et al., 1996; Fornerod et al., 1997). In the cytoplasm, importin β forms a complex with cargo proteins displaying NLS (directly or through the adaptor importin α) and moves actively into the nucleus through the nuclear pores. This process involves the interaction of importin β with the phenylalanine/glycine repeat domains displayed by nucleoporins of the NPC to overcome the size limit of the barrier and to rapidly cross the NE (Joseph, 2006; Matchett et al., 2014). In the nucleus, importin β interacts with Ran-GTP, allowing the dissociation of the complex and the release of imported proteins (Joseph, 2006; Matchett et al., 2014). Ran-GTP/importin β complex is then exported to the cytoplasm where Ran-GTP is converted into Ran-GDP, leading to the release of importin β that becomes available for another round of protein import. For the protein export process, nuclear Ran-GTP interacts with exportins together with their cargo carrying a NES and cross the NE. Once in the cytoplasm, Ran-GTP is converted into Ran-GDP, leading to the dissociation of the complex and the release of exported proteins (Joseph, 2006; Matchett et al., 2014). Cytoplasmic Ran-GDP is then translocated to the nucleus by nuclear transport factor 2 (NTF2) where it is loaded with GTP (Ribbeck et al., 1998). During mitosis, Ran-GTP promotes spindle assembly through the release of TPX2 (Targeting Protein for Xklp2) in close proximity to the chromosomes and regulates microtubule organization and dynamics (Gruss et al., 2001). At the telophase, Ran regulates the NE formation by vesicle fusion and by the assembly of the nuclear pore complex [reviewed in Joseph (2006) and Matchett et al. (2014)].

The deregulation of Ran in cancer has been reported in several tissue types (Azuma et al., 2004; Li et al., 2006; Ouellet et al., 2006; Xia et al., 2008b; Fan et al., 2013; Caceres-Gorriti et al., 2014; Matchett et al., 2014; Schnepp et al., 2015). Furthermore, a growing body of literature places Ran as a master player of cell transformation and tumor progression as well as a promising therapeutic target. In the present review, we highlight the prognostic value of Ran GTPase in cancer patients and focus on its role in the tumorigenic process. In particular, we examine the involvement of Ran in tumor progression and metastasis, and we provide insights on the use of this GTPase as a therapeutic target in cancer.

## Clinical Relevance of Ran GTPase

As discussed in detail in the following section, Ran is involved in different processes associated with cancer initiation and/or progression. Here we detail studies that have monitored its expression in clinical samples and correlated this expression with patient outcomes.

**FIGURE 1:**
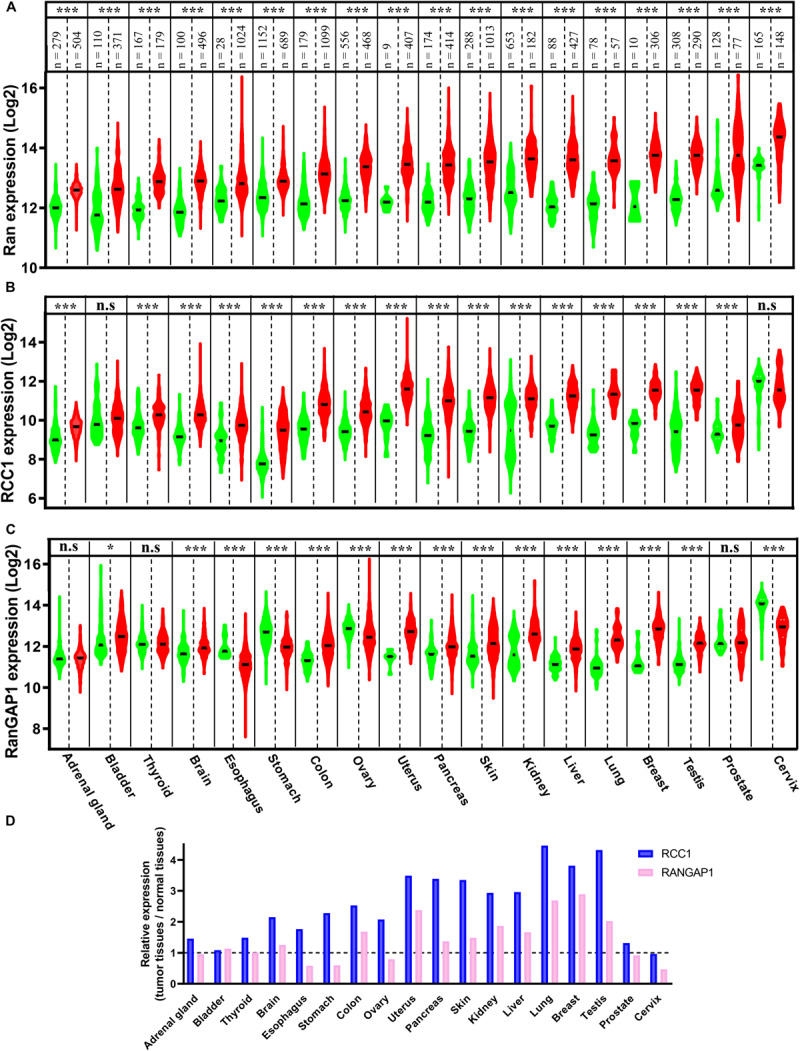
The expression of Ran, Regulator of Chromosome Condensation 1 (RCC1), and RanGAP1 in normal and tumor tissues. The expression of Ran **(A)**, RCC1 **(B)**, and RanGAP1 **(C)** in normal (green) and tumor tissues (red) was extracted from the Xenabrowser web site. *p < 0.05, ***p < 0.001 (Student’s *t*-test). **(D)** The expression of RCC1 (blue) and RanGAP1 (pink) in cancer tissues was expressed as the fold change over normal tissues.

Ran has been found to be a prognostic factor of myeloma, lymphoma, neuroblastoma, and renal cell, ovarian, and breast carcinomas (Harousseau et al., 2004; Ouellet et al., 2005, 2006; Abe et al., 2008; Hartmann et al., 2008; Schnepp et al., 2015; Sheng et al., 2018). Furthermore, among these cancers, Ran has been found to be associated with higher grades, local invasion, and metastasis in renal, breast, and ovarian cancers (Ouellet et al., 2006; Abe et al., 2008; Sheng et al., 2018). Apart from its prognostic value, in comparison with normal tissue counterparts, the expression of Ran was found to be increased in breast, renal, gastric, colon, pancreatic, ovarian, and lung cancers (Azuma et al., 2004; Ouellet et al., 2005, 2006; Abe et al., 2008; Xia et al., 2008b; Yuen et al., 2012; Deng et al., 2014; Sheng et al., 2018). Interestingly, by interrogating the Xena Functional Genomics Explorer, which allows the comparison of gene expression in tumors and normal tissues of several cancers^1^, we found that the expression of Ran was increased not only in the above mentioned cancers but also in all available cancer types, including brain, bladder, adrenal gland, thyroid, esophageal, uterine, liver, testicular, prostate, and cervical cancers (Figure 1A). Furthermore, by analyzing the expression of two essential partners of Ran involved in GTP loading (RCC1) and hydrolysis (RanGAP1), we found that while the RCC1 gene is clearly overexpressed in 16 of 18 studied cancers (Figure 1B), the dysregulation of RanGAP1 is cancer dependent (Figure 1C). Finally, by analyzing the change in gene expression between normal and transformed tissue in each cancer, we found that tumors are characterized by an imbalance of RCC1 and RanGAP1 in favor of Ran activation (Figure 1D). Overall, these observations not only reinforce the involvement of Ran in cancer initiation and progression but also should stimulate interest in the involvement of this GTPase in other cancers for which Ran is poorly investigated.

## Involvement of Ran GTPase in Cancer Progression

Cancer cells evolve through a process during which they accumulate mutations and epigenetic modifications allowing them to acquire several biological capabilities, termed the hallmarks of cancer (Hanahan and Weinberg, 2011). In this section, we investigate the contribution of Ran in the acquisition of three of them, notably, proliferative signaling, resisting cell death, and activating pathways that support invasion and metastasis.

### Proliferative Signaling

**FIGURE 2:**
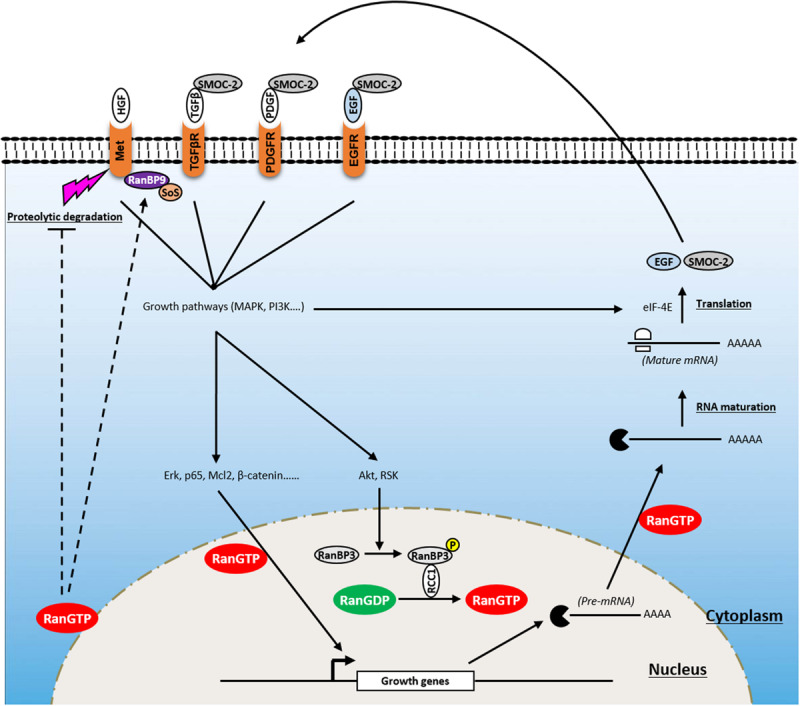
Involvement of Ran in proliferative signaling of cancer cells. Ran is a key player in a positive feedback loop that enhances growth signaling and promotes tumorigenesis. Owing to its ability to affect the import of critical transcription factors (i.e., Erk, p65, Mcl2, β-catenin), RanGTP transmits growth signals from the cytoplasm to the nucleus. RanGTP is also involved in the export of pre-mRNA which are further processed and translated into growth factors [i.e., epidermal growth factor (EGF), SMOC-2) which activate several tyrosine kinase receptors [i.e., EGF receptor (EGFR), platelet-derived growth factor receptor (PDGFR), transforming growth factor beta receptor (TGFβR)] after their release to the extracellular compartment. In addition, Ran is activated following the induction of growth signaling through the phosphorylation of RanBP3 by Akt and ribosomal protein S6 kinase (RSK). Furthermore, besides its ability to modulate the activity of these receptors, Ran is involved in supporting Met signaling through (i) its stabilization by blocking its proteolytic cleavage by metalloproteases and preventing receptor shedding and (ii) the activation of RanBP9 that recruits Son of Sevenless (SoS) which acts upstream of the GTPase Ras.

Under physiological conditions, cell proliferation is tightly controlled and occurs in response to environmental stimuli. This process controls the homeostasis of cell numbers and maintains tissue architecture and function. Proliferative signaling involves growth factors [i.e., epidermal growth factor (EGF), platelet-derived growth factor (PDGF)], their cognate receptors, often displaying tyrosine kinase activity [i.e., EGF receptor (EGFR), PDGF receptor (PDGFR), Met-receptor], and signaling pathways [i.e., mitogen-activated protein kinase (MAPK), phosphoinositide 3-kinase (PI3K), Janus kinase (JAK)/signal transducer and activator of transcription (STAT)3], which are controlled by these receptors. During cell transformation, cancer cells acquire the ability to constitutively activate these pathways, allowing aberrant cell growth which initiates the formation of tumor lesions. In this section, we discuss the role of Ran in inducing cell transformation and tumor initiation through the activation of proliferative pathways (Figure 2).

To date, no naturally occurring activating mutation of Ran has been identified. However, a number of “artificial Ran-activated mutants” have been developed including RanF35A, RanQ69L, and RanG19V (Ren et al., 1995; Lounsbury et al., 1996; Ly et al., 2010). Using immunofluorescence approaches, it has been reported that while wild-type (WT) Ran was localized predominantly in the nucleus, Ran mutants localized mainly on the NE (Lounsbury et al., 1996; Khuperkar et al., 2015). Interestingly, it was shown that ectopic expression of one of them (RanF35A) is able to transform fibroblast cells, which are then able to form tumors in mice, demonstrating that Ran activation is sufficient for cell transformation and tumor initiation (Ly et al., 2010). This was also true for already transformed cells (such as breast cancer SKBR3 cells), where the activation of Ran accentuates their transformation state (Milano et al., 2012). RanF35A overexpression is associated with the stimulation of cell growth under low serum conditions, loss of contact inhibition, and induction of anchorage-independent growth in soft agar. Furthermore, it was reported that the stimulation of cell growth by heregulin or serum induces Ran activity, which in turn participates in the translocation of transcription factors [such as Mcl-2, p65 nuclear factor-κB (NFKB), β-catenin, extracellular signal-regulated kinase (ERK), transforming growth factor-β (TGF-β)] involved in the growth response from the cytoplasm to the nucleus (Ly et al., 2010; Chandra et al., 2012; Yuen et al., 2012; Maik-Rachline et al., 2019). It also promotes shuttling of capped pre-mRNA from the nucleus to the cytoplasm to be further processed and translated by eIF-4E (Ly et al., 2010). Ran activation following serum stimulation appears to be mediated by the PI3K/Akt/mammalian target of rapamycin (mTOR) and Ras/mitogen-activated protein kinase kinase (MEK)/ERK pathways since specific inhibitors of these pathways impair the ability of Ran to induce growth in low serum conditions and in soft agar (Ly et al., 2010) and limit the extensive apoptosis seen with Ran knockdown alone (Yuen et al., 2012). Furthermore, it has been reported that the MAPK and PI3K pathways may influence the activation of Ran through ribosomal protein S6 kinase (RSK) and Akt, respectively. Mechanistically, Akt and RSK bind and phosphorylate RanBP3 at Ser58, which in turn activates RCC1, leading to the loading of Ran with GTP (Yoon et al., 2008). Interestingly, it was also shown that the expression of RanF35A in low serum conditions (i.e., in the absence of growth factors) is sufficient to induce the expression of growth factors such as EGF and SMOC-2 and to activate EGFR and its downstream proliferative pathways, Ras, MAPK, and PI3K pathways (Ly et al., 2010; Milano et al., 2012). Thus, owing to its role in the traffic of molecules between the cytoplasm and the nucleus, Ran is a key player of a positive feedback loop that enhances growth signaling and promotes tumorigenesis.

Furthermore, Ran knockdown experiments in breast cancer cell lines indicate that Ran might also influence the expression of the Met-receptor, another tyrosine kinase-coupled receptor involved in cell growth (Yuen et al., 2016). It has been shown that Ran knockdown impairs the activation of the PI3K pathway and cell proliferation following hepatocyte growth factor (HGF) stimulation (a cognate ligand of the Met-receptor) and diminishes gefitinib resistance mediated by Met-receptor overexpression. Although it is not clear how Ran regulates the expression of Met-receptor, it seems that this process occurs at the posttranscriptional level through the inhibition of a metalloprotease involved in receptor shedding (Yuen et al., 2016). Moreover, it has been shown that in breast cancer tissues, the expression of Ran is associated with that of the Met-receptor and that the combination of these two proteins has a prognostic value. Another study has shown that RanBP9, a presumed interacting partner of Ran (Nakamura et al., 1998), is an adaptor of the Met-receptor that allows the recruitment of the Son of Sevenless (SoS) protein (acting upstream of the GTPase Ras), resulting in the activation of the proliferative MAPK signaling pathway (Wang et al., 2002). Overall, these findings suggest that Ran may regulate the PI3K and MAPK pathways at two levels: upstream by stabilizing Met-receptor and recruiting SoS protein through RanBP9 and downstream by ensuring the traffic of transcription factors controlled by these pathways.

### Resisting Cell Death

**FIGURE 3:**
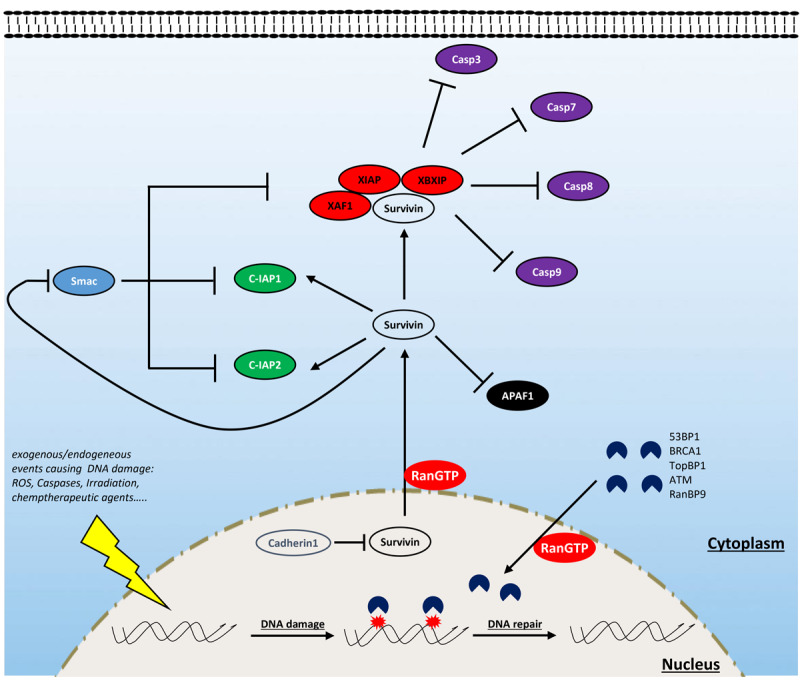
Involvement of Ran in resisting cell death. Ran is involved in resisting cell death through the interaction with survivin and some components of the DNA damage response (DDR). Ran participates in the export of survivin from the nucleus (which is rapidly destabilized by cadherin 1) to the cytoplasm. Cytoplasmic survivin plays a critical role in cell survival by inhibiting proapoptotic factors: while it participates in the formation of a complex with some inhibitors of apoptosis proteins (IAPs) (notably XAF1, XIAP, and XBXIP) to inhibit the activity of caspases 3, 7, 8, and 9, it enhances the activity of other factors such as C-IAP1 and C-IAP2. Furthermore, survivin antagonizes the release of apoptotic protease-activating factor 1 (APAF1) from the mitochondria and constrains the action of the IAP inhibitor Smac. Ran is also an active player of the DDR which is activated following the exposure of cells to endogenous or exogenous DNA-damaging factors [i.e., reactive oxygen species (ROS), caspases, irradiation, and chemotherapy]. During this process, Ran participates in the import of critical components of the DDR such as ATM, BRCA1, TopBP1, and 53BP1.

During tumor proliferation and progression, cancer cells are exposed to several apoptotic signals that are continuously induced by endogenous (i.e., oncogene signaling, DNA damage associated with aberrant proliferation) and exogenous (i.e., hypoxia, chemotherapeutic agents) stress. To overcome this, cancer cells often disrupt the balance between proapoptotic and antiapoptotic factors to maintain cell survival in conditions of extreme stress. In this section, we discuss the role of Ran in supporting the antiapoptotic protein survivin and the newly identified role of this GTPase in bypassing senescence through DNA damage response (DDR) regulation under stress conditions (Figure 3).

Survivin is of particular interest in the field of oncology since, in comparison with normal tissues, it is among the most overexpressed proteins in cancer (Jaiswal et al., 2015). Depending on its subcellular localization, survivin contributes to tumor progression by two different ways: supporting proliferation and resisting cell death (Wheatley and Altieri, 2019). The role of survivin in cell proliferation is attributed to its ability to support mitosis in highly proliferating cancer cells since its removal is accompanied by severe mitotic defects, cell cycle arrest, and apoptosis (Yang et al., 2004). Survivin targets the chromosomal passenger complex to the centromeres and, in cooperation with aurora-B kinase, ensures the alignment of chromosomes before their segregation during anaphase (Wheatley et al., 2001, 2007; Carvalho et al., 2003; Lens et al., 2003). During interphase, survivin is absent in normal cells, but in cancer cells, it is stabilized and relocalized to the cytoplasm and mitochondria where it exerts its antiapoptotic effect (Wheatley and Altieri, 2019). In the cytoplasm, survivin is a scaffold protein that interacts with inhibitors of apoptosis proteins (IAPs) to inhibit the effect of caspases 3, 7, 8, and 9 (Verhagen et al., 2001; Marusawa et al., 2003; Wheatley and Altieri, 2019). Furthermore, survivin also antagonizes both the release of apoptotic protease-activating factor 1 (APAF1) from the mitochondria and the action of the IAP inhibitor Smac (Song et al., 2004).

Interestingly, the activity of survivin, in both mitosis and apoptosis, has been reported to be strongly dependent on Ran. At mitosis, survivin was shown to regulate microtubule dynamics, which is mediated by Ran and TPX2, and the loss of Ran results in mitotic defects similar to that of survivin inhibition (Rosa et al., 2006; Xia et al., 2008b; Cheung et al., 2009). Survivin, being a small protein of 16 kDa without an NLS sequence, has a passive import through the nuclear pores, independent of the classical Ran/importins pathway (Wheatley and Altieri, 2019). However, the export of this protein is well studied, and it is closely regulated by Ran and exportin1 (XPO1) (Stauber et al., 2007). In fact, the use of an exportin inhibitor results in a strong accumulation of survivin in the nucleus, and nuclear survivin fails to protect tumor cells against chemo- and radiotherapy-induced apoptosis (Knauer et al., 2007). Thus, it is likely that Ran is a key player for the cytoplasmic localization of survivin and therefore participates in the acquisition of the antiapoptotic property of cancer cells. Although Ran knockdown was shown to be associated with decreased survivin expression in breast and pancreatic cancer cells (Xia et al., 2008b; Deng et al., 2013), the mechanism behind this regulation remains unclear. It is plausible that the export of survivin from the nucleus by the Ran/XPO1 complex contributes indirectly to the stabilization of this protein since, in contrast to cytoplasmic survivin, nuclear survivin is quickly degraded through a process involving cadherin 1 (Connell et al., 2008). Furthermore, overexpression of survivin rescued the effect of the loss of Ran in MCF7 breast cancer cells, suggesting the pivotal role of a Ran/survivin axis in breast cancer survival (Xia et al., 2008b).

Senescence is another mechanism of cell defense which is assumed to be a barrier to tumorigenesis that may operate independently or in cooperation with apoptosis. Briefly, senescence is a state during which cells enter into an irreversible non-proliferative but viable state (Collado and Serrano, 2010; Hanahan and Weinberg, 2011). During this process, senescent cells undergo a morphological change (enlarged cytoplasm) and express the senescence-associated β-galactosidase enzyme. This phenotype is induced by the activation of oncogenes or by the accumulation of DNA damage associated with a high rate of proliferation or the action of therapeutic agents (such as chemotherapy and radiation) (Zeng et al., 2018). Interestingly, the activation of Ran by the overexpression of RCC1 leads to a bypass of this senescent phenotype and cells resume their cell cycle even in the presence of the chemotherapeutic agent doxorubicin (Cekan et al., 2016). Importantly, in this study, the authors show that the overexpression of RCC1 in colorectal carcinoma cells confers an obvious resistance to doxorubicin, while the inhibition of this protein renders these cells sensitive. Mechanistically, it was shown that Ran facilitates DNA damage repair caused by doxorubicin treatment through the nuclear import of the important DDR component 53BP1 by the Ran/importin-β pathway (Cekan et al., 2016). This study is the first to argue for the involvement of Ran in DDR. However, given the large size of DDR proteins, it is likely that other components of the DDR system also require an active Ran-dependent import system to cross the NE and to induce DNA repair. In fact, some reports have shown the involvement of this GTPase in the traffic of other critical DDR proteins such as ataxia telangiectasia mutated (ATM), breast cancer gene 1 (BRCA1), and TopBP1 (Thompson, 2010; Moudry et al., 2012; Bai et al., 2014; Cekan et al., 2016; Dworak et al., 2019). However, whether modulating Ran is an interesting avenue to circumvent resistance to DNA-damaging therapies remains poorly studied. Furthermore, recent studies have shown that RANBP9 is involved in DDR [reviewed in Palmieri et al. (2018)]. An unbiased study analyzing 533 genetically annotated human cancer cell lines after radiation exposure has shown that the mutation of the RANBP9 gene is closely associated with radiation sensitivity (Yard et al., 2016). Mechanistically, RANBP9 is phosphorylated upon genotoxic stress [irradiation, ultraviolet exposure, cisplatin, and osmotic shock (Denti et al., 2004; Gong et al., 2009; Palmieri et al., 2016)] and shuttles between the cytoplasm and the nucleus (Gong et al., 2009; Palmieri et al., 2016). In particular, it has been shown that upon irradiation, RANBP9 is phosphorylated by ATM [one of the most important kinases of the homologous recombination (HR) DNA repair pathway] and localized in the nucleus and, conversely, that knockdown of RANBP9 reduces HR and induces senescence following irradiation exposure (Palmieri et al., 2016).

Overall, these data suggest that Ran may regulate DNA repair through at least two mechanisms (nucleocytoplasmic trafficking and RANBP9 interaction) and highlight the need for further investigations to elucidate this new function.

### Activating Invasion and Metastasis

**FIGURE 4:**
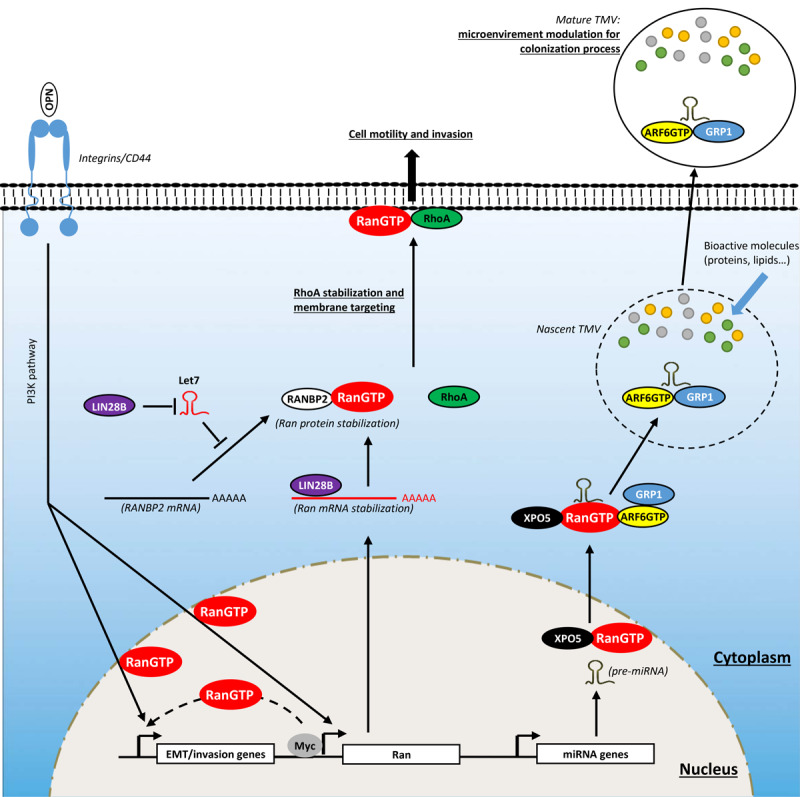
Involvement of Ran in activating invasion and metastasis. Ran, through direct and indirect interactions with oncogenes, is involved in cell invasion. Myc, which is overexpressed in several cancers, interacts directly with the promoter of Ran and induces its expression. The LIN28B oncogene stimulates the expression of Ran directly through the stabilization of its mRNA by direct interaction and indirectly the destabilization of Let7 which is known to destabilize Ran protein through RANBP2 degradation. The induction of Ran promotes cell invasion by different ways: (1) by inducing epithelial-to-mesenchymal transition (EMT); (2) by mediating the invasive signals originating from osteopontin (OPN), Myc, and LIN28B; (3) by stabilizing and targeting RhoA to the plasma membrane; and (4) by delivering oncogenic cargo such as pre-miRNAs to nascent tumor-derived microvesicles (TMVs).

Metastasis is a multistep process during which carcinoma cells undergo a morphological change called epithelial-to-mesenchymal transition (EMT) characterized by the loss of cell–cell contact with concomitant migration ability. To undergo EMT, cancer cells reactivate embryogenic programs that activate EMT-associated transcription factors including Snail, Slug, Twist, and Zeb1/2, which are involved in the reprogramming of adhesion molecules expressed by cancer cells (Micalizzi et al., 2010; Nieto, 2013). As in organogenesis, this reprogramming involves the loss of E-cadherin molecules in favor of N-cadherin (Micalizzi et al., 2010; Nieto, 2013). These changes, together with others (expression of matrix-degrading protease, increased motility, and resistance to apoptosis), allow cancer cells to invade the tumor microenvironment, to reach blood and/or lymphatic vessels (intravasation process), to invade distant tissue (extravasation process), and to form micrometastatic lesions which will grow into macroscopic tumors (colonization process) (Hanahan and Weinberg, 2011). In this section, we present some reports showing the involvement of Ran in mediating invasive/metastatic signals emitted by oncogenes and how Ran intervenes at different levels in this process (Figure 4).

It had been reported that Ran overexpression in breast and lung cancer cell lines is associated with a significant increase in cell invasion (Kurisetty et al., 2008; Ning et al., 2013). More importantly, the overexpression of Ran in a benign breast cancer model (Rama 37) was sufficient to confer an aggressive *in vivo* phenotype with 50% of these tumors being able to form metastases in lung and lymph nodes (Kurisetty et al., 2008). Conversely, Ran knockdown in a model of pancreatic cancer is associated with a significant decrease in the number of liver metastases (Deng et al., 2014). This highlights the pivotal role of Ran in cancer aggressiveness and metastasis. Moreover, Ran has been shown to be a key player to mediate signaling pathways originating from well-documented cancer metastasis promoters such as the glycophosphoprotein osteopontin (OPN) [reviewed in Zhao et al. (2018)], the RNA-binding protein LIN28B (Balzeau et al., 2017; Lu et al., 2018; Yong et al., 2018; Zhang et al., 2018, 2019), and the oncogene c-Myc (Wolfer et al., 2010; Wolfer and Ramaswamy, 2011; Dang, 2012).

In particular, Kurisetty et al. (2008) have shown that the overexpression of OPN in a breast cancer model increased *in vitro* adhesion and invasion to fibronectin-coated Boyden chambers and the number of lung metastases *in vivo*. Interestingly, the authors observed that the overexpression of OPN was concomitant with the induction of Ran at the mRNA and protein levels, and that the inhibition of Ran using specific siRNA totally abrogated the effect of OPN *in vitro* and *in vivo* (Kurisetty et al., 2008). Although there is no insight on how OPN regulates the expression of Ran, this study provides strong arguments suggesting that the oncogenic effect of OPN is largely mediated by Ran. Furthermore, in another study, a significant correlation between the expression of OPN and Ran was reported using samples from pancreatic cancer patients (Saxena et al., 2013).

LIN28B is an RNA-binding protein known as an emerging oncogenic driver in several cancers. Its oncogenic role is attributed essentially for its ability to, respectively, stabilize and destabilize oncogenic mRNA and long non-coding RNAs (LncRNAs), and tumor suppressor miRNAs. Initially, its oncogenic role was attributed to the ability to destabilize the tumor suppressor miRNA let7 [reviewed in Balzeau et al. (2017)]. However, recent studies have shown that LIN28B may act independently from let7. In ovarian cancer, LIN28B stabilizes the lncRNA NEAT1, which in turn sequesters miR506 [a suppressor of EMT through the inhibition of vimentin, zinc-finger E-box binding homeobox (ZEB)1, avian erythroblastosis virus E26 homolog-1 (ETS1), Rho-associated kinase (ROCK)1, and zinc finger protein SNAI1 (SNAIL)] to induce EMT and cell migration (Li et al., 2015, 2017; Sun et al., 2015; Yong et al., 2018). Furthermore, irrespective of its role in cell invasion, LIN28B displays an antiapoptotic role in ovarian cancer cells through the regulation of the protein kinase Bβ (AKT2)/forkhead box O3a (FOXO3A)/Bcl-2-like 11 (BIM) axis (Lin et al., 2018). In lung cancer cells, LIN28B stabilizes the mRNA of Delta-like protein 3 and induces cancer cell proliferation and migration (Huang et al., 2019). It also promotes EMT and invasion through the induction of interleukin (IL)-6 release and STAT3 phosphorylation (Lu et al., 2018). In colorectal cancer cells, LIN28B is involved in cancer progression by stabilizing the mRNA of the oncogenic insulin receptor substrate 1 (Tang et al., 2019). In gastric cancer, LIN28B promotes cancer cell stemness by stabilizing neuropilin 1 mRNA and activating the downstream Wnt/β-catenin signaling (Luo et al., 2016; Wang et al., 2018b). Schnepp et al. (2015) have shown a strong correlation between the expression of Ran and LIN28B in samples from patients with neuroblastoma, particularly those with *MYC* amplification. Furthermore, loss of LIN28B in neuroblastoma cells is associated with a significant decrease in Ran expression both at the mRNA and protein levels. Mechanistically, it was shown that LIN28B supports the expression of Ran in two ways: (1) directly by interacting and stabilizing Ran mRNA and (2) indirectly through the degradation of let-7 miRNA, which is a negative regulator of RANBP2 [itself found to stabilize Ran protein (Patil et al., 2014)] (Schnepp et al., 2015). Importantly, the same study showed that the loss of the oncogenic effect of LIN28B by shRNA is totally rescued by the overexpression of Ran. This highlights the pivotal role of Ran in mediating oncogenic LIN28B signaling.

In regard to the oncogene Myc, Yuen et al. (2013) have shown that the overexpression of Myc is associated with the expression of Ran, and conversely, Myc silencing is accompanied by decreased expression of this GTPase. Mechanistically, it was revealed that Myc interacts directly with the promoter of Ran (68 base pairs upstream of the translational initiation site) and induces its expression (Yuen et al., 2013). As for OPN and LIN28B, Ran knockdown also reversed the effect induced by Myc overexpression, suggesting the importance of Ran in mediating the oncogenic effect of Myc in breast cancer. Clinically, Ran expression is correlated with that of Myc in lung and breast cancer patient samples (Yuen et al., 2013). Moreover, in breast and lung tumors overexpressing Myc, Ran was shown to be a potent biomarker, where overexpression is seen in the most aggressive cases (Yuen et al., 2013). In summary, Ran is crucial for mediating signals originating from oncogenes known to induce invasion and cancer metastasis.

In the rest of this section, we provide an overview of the upstream signaling engaged by Ran to induce this aggressive phenotype (Figure 4). It has been reported that the overexpression of Ran induces EMT (increased N-cadherin and decreased E-cadherin expressions) and cell invasion in non-small-cell lung cancer cells through a PI3K-dependent and MAPK-independent pathway (Ning et al., 2013). These observations are consistent with the circuitry linking Ran to OPN and to Myc since these two oncogenes are, respectively, positioned upstream (Kurisetty et al., 2008) and downstream (Chen et al., 2018; Yang et al., 2019) of the PI3K pathway. Accordingly, Ran knockdown was shown to be associated with an inhibition of cell invasion and EMT in breast cancer cell lines since it induces E-cadherin and decreases vimentin expression (Sheng et al., 2018). Overall, the data presented here argue for a role of Ran in EMT, although how Ran may regulate this process remains unclear. A likely possibility is that Ran is involved in the nuclear import of EMT-associated transcription factors (SNAIL, SLUG, ZEB1/2) since these all retain an NLS motif.

Recently, we have shown that Ran is involved in ovarian cancer invasion through an unexpected mechanism (Zaoui et al., 2019) relying on a new role/localization of this GTPase, totally independent of its well-documented function in nucleocytoplasmic transport and mitosis. By investigating cell migration in two aggressive epithelial ovarian cancer cell lines following Ran knockdown, we observed an unusual phenotype characterized by reduced spreading and motility while producing long projections that appeared at the trailing end of cells. Since this phenotype has been observed in other models following the loss of RhoA (Worthylake et al., 2001; Vega et al., 2011; Bzymek et al., 2016), it raised the question whether Ran and RhoA cooperate to induce cell migration and invasion. RhoA is a member of the Rho family that has been extensively studied for its role in cell migration and invasion. Following an oncogenic stimulation, RhoA is recruited to the cell membrane in specialized structures and participates in several steps in cell motility and invasion [reviewed in Ridley (2001)]. Surprisingly, in our study, we observed that following serum stimulation, Ran colocalizes with RhoA at the plasma membrane, particularly in the motile ruffles. Furthermore, in constructs that shuttle Ran to the mitochondria (using a mito tag), RhoA was found to be colocalized in these organelles. Mechanistically, we showed that Ran, through its DEDDDL domain, interacts with the C-terminal region of RhoA (particularly at the Ser188) and avoids its proteasome degradation (Zaoui et al., 2019). These findings together with the knowledge that several RhoA-GEFs are under the control of G protein-coupled receptors (Yu and Brown, 2015), that are involved in cancer progression (Lappano and Maggiolini, 2012), open new perspectives in the role of Ran in mediating certain membrane proximal signaling events that should be investigated in the future. Furthermore, it will be interesting in the future to determine in other cancer models how and whether Ran localizes to the plasma membrane and whether it cooperates with other new oncogenic partners.

It is now recognized that local or distant cell–cell communication is crucial for tumor pathogenesis and metastasis (Desrochers et al., 2016). This process largely involves tumor-derived microvesicles (TMVs) (Desrochers et al., 2016). These vesicles are generated through a budding from the plasma membrane into the extracellular environment where they can interact and influence the behavior of neighbor or distant recipient cells (Abels and Breakefield, 2016; Tricarico et al., 2017). Bioactive cargos that can be transported by these vesicles include proteases, cell surface receptors, active lipids, and miRNAs (Melo et al., 2014; Clancy et al., 2015; Menck et al., 2015; van Niel et al., 2018). Interestingly, it has been reported that the capture of miRNA-loaded vesicles by non-invasive tumor cells promotes metastatic colonization of these cells, suggesting the involvement of miRNA-loaded vesicles in tumorigenesis and metastasis (Zhang et al., 2015). In this regard, Ran was shown to be indirectly linked to the TMV biogenesis in melanoma, breast, and prostate cancer models, particularly for loading these nascent structures with miRNAs (Clancy et al., 2019). This process involves the formation of a complex Ran-GTP/exportin-5 (XPO5)/pre-miRNA which after nuclear export interacts with and delivers pre-miRNAs to the shuttle ADP-ribosylation factor 6 (ARF6)-GTP/general receptor of phosphoinositides 1 (GRP1) which loads these miRNAs into the TMV. The generated TMVs were then able to influence and transform recipient fibroblast cells (Clancy et al., 2019). Importantly, these transformed fibroblasts overexpressed α-SMA, indicating the acquisition of a myofibroblast phenotype which is often associated with metastasis (Karagiannis et al., 2012; Khamis et al., 2012). Although it appears that Ran is indirectly involved in this process, this study pinpoints the identification of a new complex in which the GTPase ARF6 and Ran cooperate to influence the tumor microenvironment. Another study has shown that Ran itself, particularly the active form, can be transferred between donor and recipient cells (Khuperkar et al., 2015); however, the mode of action and the consequences of this transfer remain to be elucidated. Overall, these observations open new perspectives for future investigations on the role of Ran in modulating the tumor microenvironment.

## Therapeutic Targeting of Ran GTPase

We and others have shown that Ran inhibition using siRNA/shRNA is toxic for cancer cells both *in vitro* and *in vivo*, irrespectively of their origin or genetic background (Barres et al., 2010; Yuen et al., 2012, 2013; Deng et al., 2014; Schnepp et al., 2015). However, the loss of Ran was shown to be well tolerated by different normal cells (Xia et al., 2008a, b; Deng et al., 2013). This data together with the many reports showing the overexpression of Ran in cancer cells (Azuma et al., 2004; Ouellet et al., 2005, 2006; Abe et al., 2008; Xia et al., 2008b; Yuen et al., 2012; Deng et al., 2014; Sheng et al., 2018) and our analysis (Figure 1A) showing that normal cells have lower Ran mRNA expression than cancer cells suggest that Ran would be a promising therapeutic target for several cancers. Here we present recent studies that aim to target Ran in the context of cancer therapy.

Due to the success of the siRNA/shRNA technology on *in vitro* and xenograft studies, many efforts have been made to use this technology clinically. However, an effective delivery of these negatively charged nucleotides is a challenge since they poorly cross the plasma membrane of target cells (Leng et al., 2009). Recently, conjugation of siRNA/shRNA with poly(lactide-co-glycolide) (PLGA) nanoparticles (NPs) was shown to be effective to circumvent this delivery problem both *in vitro* and *in vivo* (Wang et al., 2017; Risnayanti et al., 2018). In this regard, Sharma et al. (2018) have shown the effectiveness of encapsulating two shRNAs targeting Ran in PLGA-NPs and demonstrated their ability to deliver the shRNAs and to knockdown the expression of Ran in a model of triple-negative breast cancer (TNBC). As expected, Ran knockdown caused a significant decrease in cell survival and in the invasion ability of breast cancer cells (Sharma et al., 2018). This approach provides a new strategy to target Ran that still needs to be assessed in preclinical *in vivo* xenograft models. Another study has shown that miR203 interacts with Ran mRNA at its 3′UTR region leading to the suppression of its expression (Zhang et al., 2014). Interestingly, miR203 has been shown to display a tumor-suppressive function and to be downregulated in the context of esophageal cancer (Fassan et al., 2011; Yuan et al., 2011). Further functional investigations in an esophageal cancer cell line revealed that miR203 inhibits cell proliferation and invasion and induces apoptosis partly through the inhibition of Ran (Zhang et al., 2014; Xia et al., 2019). Importantly, it has been shown *in vivo* that intratumoral injection of a miR203 mimic is able to inhibit tumor growth (Xia et al., 2019). Since NP technology could be applied for microRNA delivery (Lee et al., 2019), it is conceivable that the use of miR203 as an anticancer therapy would be effective.

In a recent publication, Dakir et al. (2018) demonstrated that pimozide, a potent antagonist of the dopamine receptor D2 (D2R), was able to inhibit the expression of Ran at the transcriptional level. This molecule was tested in TNBC and lung cancer cell lines, MDA MB231 and A549, respectively, and showed potent toxicity in these cells without any effect in the non-transformed MCF10A cells. Importantly, Pimozide delayed *in vivo* tumor growth and significantly reduced the number of lung metastases. Whether the modulation of Ran by Pimozide is mediated through the D2R remains to be investigated. The authors did show that Pimozide treatment represses the expression of c-Myc, known to interact with the promoter of Ran to induce its expression (Yuen et al., 2013). As pimozide is already used in the clinic as an antipsychotic for patients with schizophrenia and other psychotic disorders, clinical trials with this molecule could be undertaken quickly. Importantly, preclinical studies were done at higher doses than are presently used in humans so that there remains a challenge to find an effective but well-tolerated dose in the context of cancer since D2R is widely expressed in the brain and the repression of this receptor may cause severe side effects. In fact, it has been shown that mice lacking D2R demonstrate an impairment of motor activity and movement coordination as well as hyperactivity (Anzalone et al., 2012).

In order to target the activity of Ran rather than its expression, there have also been reports that focus on disrupting the interaction between Ran and its cognate GEF, RCC1. To do this, the researchers identified and synthetized peptides from the Ran protein that are predicted to compete with Ran GTPase for the interaction with RCC1 (Haggag et al., 2017). *In vitro*, the activity of this peptide was suboptimal due to a reduced bioavailability and poor delivery. To overcome these issues, and as for shRNAs, this group has used PLGA-NP technology and have shown that the encapsulated peptide was able to inhibit the activity of Ran in the TNBC cell line, MDA-MD-231, resulting in a dramatic decrease of cell viability, migration, and invasion (Haggag et al., 2017, 2019). Importantly, this strategy was applied *in vivo* in a breast xenograft model and showed a significant delay in tumor growth. This new strategy is interesting and should be tested in other models of breast cancer, in particular, to assess its effectiveness to treat metastatic diseases.

## Conclusion

Ran plays a major role in mitosis and the traffic of proteins between the nucleus and the cytoplasm. Owing to the high rate of cell division and the shuttle of transcription factors and transcribed mRNA in and out of the nucleus of transformed cells, these two functions are critical for cancer growth and progression. This is consistent with the fact that Ran is often overexpressed in cancer. However, while the artificial overexpression of a dominant active mutant of Ran is able to induce cell transformation, the WT Ran does not (Ly et al., 2010). So far, Ran-GTP expression has never been evaluated in a cohort of cancer tissues. Therefore, it would be of interest to evaluate, simultaneously, the expression of total Ran and its active form and to investigate their association with patient outcomes. This may improve the prognostic significance of Ran. Moreover, since no activating mutation of Ran has been reported in patient samples, this raises the question on how Ran is activated in the context of cancer. One possible explanation is that, as shown in Figure 1, the overexpression of Ran is accompanied by an imbalance between RCC1 (involved in GTP loading) and RanGAP1 (involved in GTP hydrolysis) expression leading to the activation of Ran. Furthermore, it has been shown that the nuclear import of RCC1 relies simultaneously on a classical import pathway, involving a complex of importin α3/β and Ran-GTP, and a non-classical import pathway that does not require the presence of Ran (Nemergut and Macara, 2000; Sankhala et al., 2017). Thus, it is possible that Ran may, to some extent, exert a positive control of its own activity through the traffic of RCC1. Another possible explanation comes from the observation that single-nucleotide polymorphisms (SNPs), the most common form of genetic variation, are extensive within the *RAN* gene (Wang et al., 2018a; Li et al., 2020). However, whether these SNPs may influence Ran activity or expression remains unknown and should be investigated in future studies. Among these SNPs, two (rs14035 C>T and rs3803012 A>G) have been investigated in a meta-analysis study and were associated with cancer incidence (Li et al., 2020). Since these two SNPs are located in the 3′UTR region of Ran, this suggests that they would be involved in the regulation of the expression of Ran through the (de)stabilization of its coding mRNA.

To date, Ran has been studied in a few cancers including breast and ovarian cancers, however, as reported here, it is most likely that this GTPase is involved in other tumor types, and this should be further investigated. Ran is now proposed as a promising therapeutic target, and some recent studies have shown that this GTPase is druggable. In fact, targeting Ran would be effective to attenuate at least three hallmarks of cancer without affecting healthy cells.

The specific sensitivity of cancer cells to the loss of Ran might be explained by the theory of oncogene addiction of cancer cells (Weinstein, 2002; Weinstein and Joe, 2008). Briefly, cancer cells evolve through a process during which they accumulate mutations and epigenetic modifications in several genes that have diverse functions. However, silencing of only one or a few genes can irreversibly inhibit cancer growth and lead to cell death (Weinstein, 2002; Weinstein and Joe, 2008). In fact, in many cancer models, the removal of c-Myc, H-Ras, K-Ras, EGFR, mutant BRAF, c-Kit, or Met leads to growth arrest, differentiation, and apoptosis (Weinstein and Joe, 2008). All these oncogenes control or are under the control of the MAPK and PI3K pathways in which Ran is a critical player acting downstream of these oncogenes. This may explain why several cancer models are highly sensitive to the loss of Ran. Furthermore, it is proposed that an oncogene may play a vital and qualitatively different role in a given pathway in comparison with a normal cell. In fact, oncogenes and oncogenic pathways in normal cells are activated in a discrete or punctual manner (Weinstein and Joe, 2008). This is in accordance with the emergence of targeted therapies (targeting EGFR, MEK, cKit, mutant BRAF, Akt, mTOR) that have shown moderate toxicity in patients. This may explain why the loss of Ran is well tolerated in normal cells. However, another explanation might come from the well-known role of Ran in mitosis. In support to this idea, a report has shown that tumor cells have a steeper mitotic Ran-GTP gradient than normal cells resulting in altered pro-metaphase/metaphase timing (Hasegawa et al., 2013), which in turn can influence cell proliferation (Uetake and Sluder, 2010). Furthermore, it was also shown that this steep mitotic Ran-GTP gradient could be induced in normal human foreskin fibroblasts after the fusion of two cells, suggesting that chromosomal gain underlies the basis for this altered Ran-GTP gradient (Hasegawa et al., 2013). Hence, it is conceivable that the selective effect of Ran downregulation on tumor cells is related to aneuploidy.

Based on accumulating evidence, the therapeutic targeting of Ran might be an interesting avenue for the development of a new targeted therapy for cancer. However, it is known that developing chemical compounds competing with GTP is challenging. In fact, the affinity of a GTPase to its cognate GTP is so high that it can reach the pM range. Furthermore, the cellular concentration of GTP is very high (millimolar range), making it difficult to obtain one efficient compound with a nucleotide-competitive mode of action. However, since the crystal structure of Ran is already resolved (Scheffzek et al., 1995; Geyer et al., 1999; Monecke et al., 2009), it would be interesting in future studies to target Ran on its GDP-binding pocket and lock the GTPase in its inactive form. This strategy has already proved its worth in developing effective inhibitors against other GTPases such as Ral (Yan et al., 2014) and Arf6 (Yoo et al., 2016) and would open new perspectives for the discovery and development of new inhibitors of Ran GTPase.

## Author Contributions

ZB conceived the study, collected the material, wrote the manuscript, and generated the illustrations. EC, DP, and A-MM-M provided critical feedback and edited the manuscript.

## Conflict of Interest

The authors declare that the research was conducted in the absence of any commercial or financial relationships that could be construed as a potential conflict of interest.

## Footnotes

1 https://xenabrowser.net
